# Modelling and prediction of global non-communicable diseases

**DOI:** 10.1186/s12889-020-08890-4

**Published:** 2020-06-01

**Authors:** Yang Wang, Jinfeng Wang

**Affiliations:** 1 State Key Laboratory of Resources and Environmental Information System, Institute of Geographic Sciences and Natural Resources Research, Chinese Academy of Sciences, Beijing, 100101 China; 2 University of Chinese Academy of Sciences, Beijing, 100049 China

**Keywords:** Non-communicable diseases, Geotree, Socio-economic factors, Prediction

## Abstract

**Background:**

Non-communicable diseases (NCDs) are the main health and development challenge facing humankind all over the world. They are inextricably linked to socio-economic development. Deaths caused by NCDs should be different in different socio-economic development stages. The stratified heterogeneity of NCD deaths is currently not fully explored.

**Methods:**

Countries were classified according to their socio-economic types and development stages, which were illustrated as a tree-like structure called Geotree. NCD deaths were linked to the countries and so were attached to the Geotree, which was modelled by a multilevel model (MLM) approach. Accordingly, the levels of NCD death indexes were predicted for 2030.

**Results:**

Through the Geotree structure constructed in the study, it can be seen that the NCD death index has obvious stratified heterogeneity; that is, the NCD death index shows different trends in different country types and socio-economic development stages. In the first-level branches (country type), as national income increases, NCD mortality rate decreases and the proportion of NCD deaths to total deaths increases. In the secondary-level trunks (socio-economic development stage), as a country’s development stage rises, the NCD mortality rate decreases and the proportion of NCD deaths to total deaths increases. In addition, combined with the hierarchical nature of the evolution tree model, the MLM was used to predict the global NCD death index for 2030. The result was that by 2030, the global average age-standardized NCD mortality rate would be 510.54 (per 100,000 population) and the global average mortality for NCD deaths of the total number of deaths would be 75.26%.

**Conclusions:**

This study found that there is a significant association between socio-economic factors and NCD death indicators in the tree-like structure. In the Geotree, countries on the same branch or trunk can learn from countries with higher development stages to formulate more effective NCD response policies and find the right prevention and treatment path.

## Background

Non-communicable diseases (NCDs)—namely cardiovascular diseases, diabetes, cancer and chronic respiratory diseases—have a higher morbidity and mortality rate globally than do all other causes combined [1]. They are the main health and development challenge facing humankind in the twenty-first century. They have caused certain damage to the socio-economic structure of human beings and countries, especially low- and middle-income countries (LMICs) [1]. The distribution of NCDs was mainly widespread in developed countries in the early years. However, the NCD incidence and mortality in LMICs have been increasing in recent years [2]. It is worth mentioning that in all continents, except Africa, the number of deaths from NCDs has now exceeded the total number of deaths from communicable, maternal, perinatal and nutritional conditions [1, 3]. The World Health Organization (WHO) estimates that by 2020, NCDs will account for 80% of the global disease burden. Seven out of every ten deaths in developing countries are caused by NCDs, and about half of these deaths are in people younger than 70 years [4–7]. Moreover, the global NCD burden will increase by 17% in the next decade, and in Africa, it will increase by 27%. Nearly half of all deaths in Asia are attributable to NCDs, accounting for 47% of the global disease burden [4]. The International Symposium on “NCDs in Developing Countries” was held on 22 March 2014 at Ludwig-Maximilians-Universität in Munich, Germany, to discuss the direction of NCD control in developing countries [8]. This is an indication that developing countries are beginning to pay more attention to NCDs.

NCDs are a kind of “rich and noble diseases” in people’s conventional cognition. Their impact has been attracting attention in countries and regions with a high degree of economic development and a severely ageing population, and they are also a matter of significant concern internationally. Developed countries have been bearing the NCD burden from the early years, so they have accumulated more experience in the disease prevention and control. But, in recent years, people have discovered that NCDs are not just “rich and noble diseases”, and their impact on developing countries cannot be underestimated [9]. Developing countries and regions have not shaken off the impact of infectious diseases, and the NCD burden has increased rapidly [10]. Especially for LMICs and regions, NCDs have even become a barrier to their continued economic development and progress [11]. Ultimately, the mechanism of NCDs is inextricably linked with the influence of socio-economic factors, and the transition of human disease burden from infectious to non-infectious diseases has been driven by many factors indicating economic development [12]. It is just that the leading factors for and risks of NCDs in countries and regions at different economic development stages are different. The specific manifestations of human socio-economic development related to the occurrence of NCDs have the following major aspects: in terms of economy, increased financial capacity and increased disposable income [13–17]; in terms of diet, the transition from traditional foods to high-fat, high-salt and high-sugar processed foods, with diet structure becoming unhealthy [17–23]; in terms of work and lifestyle, because of the change in the type and nature of work and the increase in living pressure—coupled with changes in the type of daily recreational activities—people have become more sedentary and lack physical activity [16, 17, 24–29]; and in terms of educational awareness, including alcohol and tobacco consumption and control [13, 20, 25, 26, 30–40]. In addition, infectious diseases can be effectively controlled through medical means such as vaccines and drugs, but NCDs have no effective means of control because of their relatively complicated mechanism of action. Therefore, the proportion of NCD deaths will continue to increase [12]. Hence, whether in developed or developing countries, the NCD burden on human beings cannot be ignored.

This study also explores the changing trend of NCD deaths from the perspective of socio-economics, which is based on the study of socio-economic factors. Unlike previous studies, this study considers the stratified heterogeneity of socio-economic development as the entry point. The socio-economic development of different countries around the world does not necessarily follow the same path and laws, but different types show different development paths. NCD deaths are related to socio-economic development, so they may also show corresponding stratified heterogeneity. That is to say, the development trend of NCD deaths in different country types and development stages would also be different, whereas the same country types may show similar development laws. Exploring the change and development of NCD deaths on such a layered basis will get more accurate results than do ordinary global studies. The evolution tree model (Geotree) is a multidimensional visualization model with stratified heterogeneity as its core [41, 42]. The spatio-temporal evolution tree model is not limited by dimensions. By combining the development law of things, the mechanisms and evolution that may exist in multidimensional data are expressed in a simple and clear visual form [42]. In addition, the multilevel model (MLM) in Geotree is completely based on stratified heterogeneity for model fitting and predicting future changes in NCD deaths, which provides better accuracy than ordinary global models do. It is worth mentioning that Geotree not only has the results of quantitative analysis, but also covers the qualitative description, which makes it possible to explain the changing trend of NCD deaths mechanically.

## Methods

### NCD death data

There were two types of NCD death data used in this study: (i) data on NCD deaths from 176 countries in the world in 2015 and (ii) data on NCD deaths of the total number of deaths from 176 countries in the world in 2015. Both types of data were sourced from the WHO database [43]. The NCDs data used in this article mainly include diseases such as cancer, diabetes mellitus, cardiovascular diseases, digestive diseases, skin diseases, musculoskeletal diseases, and congenital anomalies.

### Socio-economic data

#### Current data

In terms of economic development, the gross domestic product (GDP) per capita was a proxy variable for a country’s level of economic development and was used to define the type of development of the country. The data for 2015 were from the World Bank database.

In terms of social development, the urbanization rate was used as an indicator to reflect the degree of social development and was used to define the development stage of a country. The data for 2015 were sourced from the World Bank database.

In terms of medical development, the neonatal infant mortality rate was used as a proxy variable for the level of medical development and was used to define the development stage of a country. The data for 2015 were from the World Bank database.

#### Future data

This study used an MLM to predict the levels of NCD deaths in countries around the world for 2030. There were two sources of socio-economic factor data used for prediction. Part of the data were from the Shared Socioeconomic Pathways (SSP) database [44–47]. The indicators used were global population data, global GDP data and global urbanization rate data.

The neonatal infant mortality data were linearly extrapolated from the 1960–2016 data (sourced from the World Bank database) to obtain neonatal infant mortality data from countries around the world for 2030 and used as explanatory variables in the MLM in prediction experiments.

### Geotree method—tree structure construction

As a complex object, the manner in which a country evolves is not completely explained by a single-model method. This complex, multidimensional development process is similar to the evolution of organisms. The evolution tree model draws on the evolutionary theory of biology, and the development path of the country can be described by the growth process of the tree structure.

Generally, the evolution tree model consists of a two-level classification. In this study, the first- and second-level classifications were the country type and country development stage, respectively. The statistical unit of the research object was 176 countries over the world, and different aspects of socio-economic factors were selected as indicators for the two-level classification. In the constructed tree structure, the first branches, second trunks and leaves of the evolution tree represented different classification information, which are the country type, development stage, and country individual, respectively. The calculation results of the evolution tree provide hierarchical heterogeneity information for subsequent research, which is a new idea to the prediction of development trend of NCD death.

#### First branches—country types

**Fig. 1:**
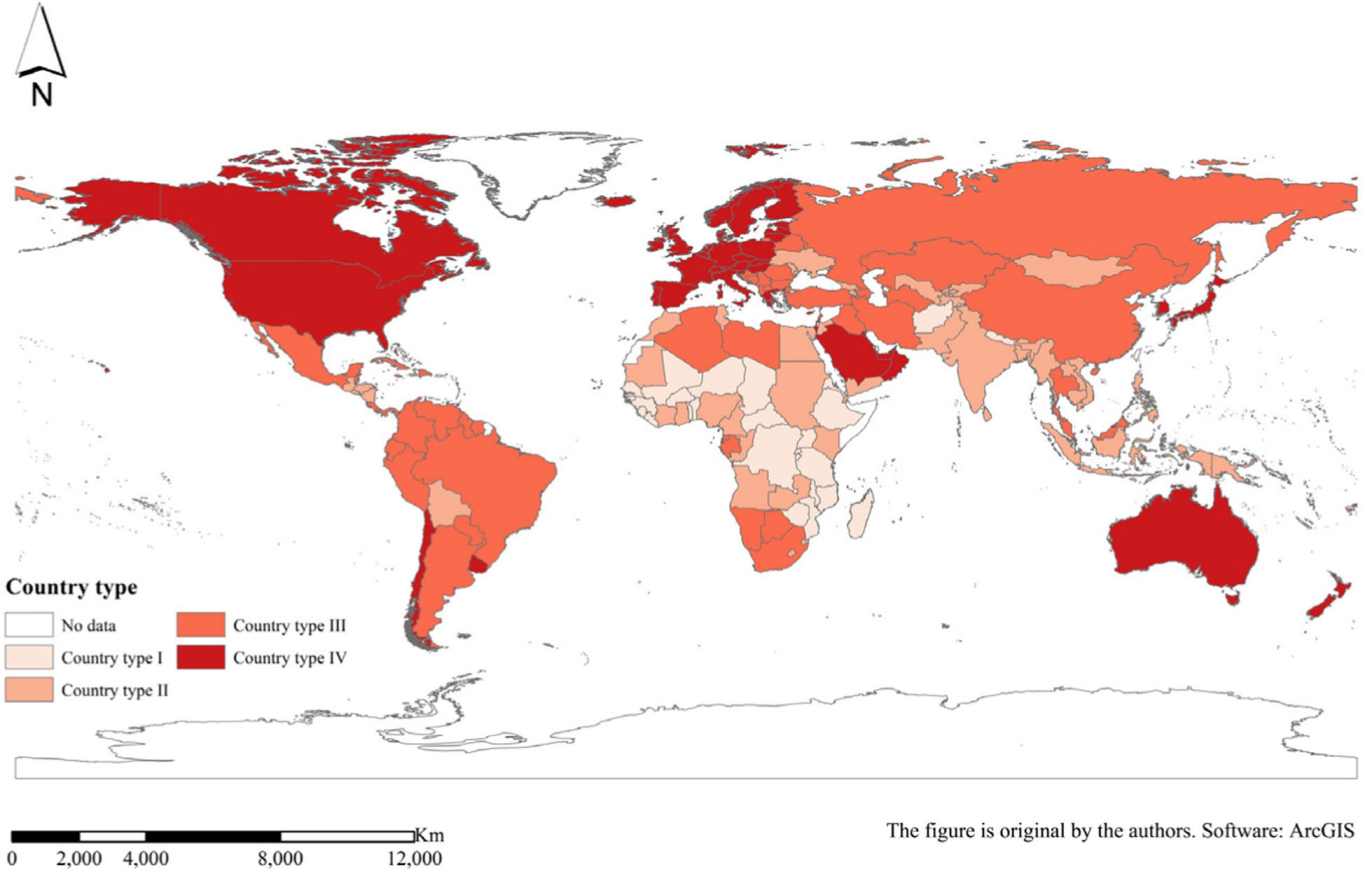
Spatial distribution of the four country types

The level of income is currently the internationally used standard for classifying countries, reflecting the overall economic development of a country and the degree of wealth of its citizens. According to the World Bank’s 2018 income standards, low-income economies are defined as those with a gross national income (GNI) per capita of $1025 or less in 2018; lower middle-income economies are those with a GNI per capita between $1026 and $3995; upper middle-income economies are those with a GNI per capita between $3996 and $12,375; and high-income economies are those with a GNI per capita of $12,376 or more. GDP per capita is roughly equivalent to GNI per capita [48], so this study divided 176 countries into four different country types on the basis of their level of GDP per capita. The four country types were (I) low-income economies (26 countries), (II) lower middle-income economies (50 countries), (III) upper middle-income economies (51 countries) and (IV) high-income economies (49 countries) (Fig. 1, Table 1).

**Table 1:** Countries belong to type I to IV

Country type	Country name
Type I	Afghanistan, Benin, Burkina Faso, Burundi, Central African Republic, Chad, Democratic Republic of the Congo, Eritrea, Ethiopia, Gambia, Guinea, Guinea-Bissau, Liberia, Madagascar, Malawi, Mali, Mozambique, Nepal, Niger, Rwanda, Senegal, Sierra Leone, Togo, Uganda, United Republic of Tanzania, Zimbabwe
Type II	Angola, Armenia, Bangladesh, Bhutan, Bolivia (Plurinational State of), Cabo Verde, Cambodia, Cameroon, Congo, Côte d’Ivoire, Djibouti, Egypt, El Salvador, Georgia, Ghana, Guatemala, Honduras, India, Indonesia, Jordan, Kenya, Kiribati, Kyrgyzstan, Lao People’s Democratic Republic, Lesotho, Mauritania, Micronesia (Federated States of), Mongolia, Morocco, Myanmar, Nicaragua, Nigeria, Pakistan, Papua New Guinea, Philippines, Republic of Moldova, Sao Tome and Principe, Solomon Islands, Sri Lanka, Sudan, Tajikistan, Timor-Leste, Tunisia, Ukraine, Uzbekistan, Vanuatu, Viet Nam, Yemen, Zambia
Type III	Albania, Algeria, Argentina, Azerbaijan, Belarus, Belize, Bosnia and Herzegovina, Botswana, Brazil, Bulgaria, China, Colombia, Costa Rica, Croatia, Cuba, Dominican Republic, Ecuador, Equatorial Guinea, Fiji, Gabon, Grenada, Guyana, Iran (Islamic Republic of), Iraq, Jamaica, Kazakhstan, Lebanon, Libya, Malaysia, Maldives, Mauritius, Mexico, Montenegro, Namibia, Panama, Paraguay, Peru, Republic of North Macedonia, Romania, Russian Federation, Saint Lucia, Saint Vincent and the Grenadines, Samoa, Serbia, South Africa, Suriname, Thailand, Tonga, Turkey, Turkmenistan, Venezuela (Bolivarian Republic of)
Type IV	Antigua and Barbuda, Australia, Austria, Bahamas, Bahrain, Barbados, Belgium, Brunei Darussalam, Canada, Chile, Cyprus, Czechia, Denmark, Estonia, Finland, France, Germany, Greece, Hungary, Iceland, Ireland, Israel, Italy, Japan, Kuwait, Latvia, Lithuania, Luxembourg, Malta, Netherlands, New Zealand, Norway, Oman, Poland, Portugal, Republic of Korea, Saudi Arabia, Seychelles, Singapore, Slovakia, Slovenia, Spain, Sweden, Switzerland, Trinidad and Tobago, United Arab Emirates, United Kingdom of Great Britain and Northern Ireland, United States of America, Uruguay

#### Second trunks—country development stages

The country development stage used a more detailed measurement of the level of social development and medical care and chose to use the urbanization and neonatal infant mortality rates as indicators for calculation. Each indicator was divided into six strata by the natural discontinuity method, and the weights were interpreted by calculating the explanatory power of influencing factors on NCD deaths, which was finally synthesized into the total development stage. The amount of explanatory power was chosen to be measured using the Geodetector *q*-statistic (Table 2). After calculation, the weights of neonatal infant mortality and urbanization rates in the secondary trunks of the NCD mortality rate evolution tree were 0.55 and 0.45, respectively. In contrast, for NCD death proportion evolution tree, the weights of neonatal infant mortality and urbanization rates were 0.75 and 0.25, respectively.

**Table 2:** *q*-Values of the second trunks indicators for NCD death indexes. (NCDs include cancer, diabetes mellitus, cardiovascular diseases, digestive diseases, skin diseases, musculoskeletal diseases, and congenital anomalies)

	Newborn infant mortality	Urbanization rate
Age-standardized NCD mortality rate (per 100,000 population)		
*q* statistic	0.46647	0.389107
*p* value	0.000	0.000
Mortality for NCDs of the total number of death (%)		
*q* statistic	0.780991	0.258799
*p* value	0.000	0.000

#### Leaves

Each of the 176 countries was classified as a country type and development stage and was represented by leaves on the trunks, which were associated with branches on the evolution tree.

### MLM method

The combination of evolution tree model and MLM acted as the key to the prediction function of the evolution tree model. The evolution tree presented a hierarchical structure that could be modelled using an MLM. The MLM extended general regression by analysing stratified [49] and cross-classified data [50, 51] while examining the effects of group- and individual-level covariates on individual-level outcomes (e.g. urban land expansion or disease rates) [52]. The MLM decomposed the total variance into different levels in the context phenomenon [53]. In addition, it operated on multiple scales or levels, so the overall model could include the microscale of the individual and the macroscale of the population [54].

#### Cross-classified MLM

Entities such as countries can be cross-classified into country types and development stages. Cross-classification allows effects from two different “backgrounds”: country type and development stage. The cross-classification model was applicable to the research object of this study, and its formula expression is as follows [55]:

$$ {\mathrm{y}}_{\mathrm{i}\left(\mathrm{t},\mathrm{s}\right)}={\upbeta}_0+{\upbeta}_1{\mathrm{x}}_{1\mathrm{i}\left(\mathrm{t},\mathrm{s}\right)}+{\mathrm{u}}_{\mathrm{t}}+{\mathrm{u}}_{\mathrm{s}}+{\mathrm{e}}_{\mathrm{i}\left(\mathrm{t},\mathrm{s}\right)} $$

1 $$ {\mathrm{u}}_{\mathrm{t}}\sim \mathrm{N}\left(0,{\upsigma}_{\mathrm{u}\left(\mathrm{t}\right)}^2\right),{\mathrm{u}}_{\mathrm{s}}\sim \mathrm{N}\left(0,{\upsigma}_{\mathrm{u}\left(\mathrm{s}\right)}^2\right),{\mathrm{e}}_{\mathrm{i}\left(\mathrm{t},\mathrm{s}\right)}\sim \mathrm{N}\left(0,{\upsigma}_{\mathrm{e}}^2\right) $$

where *y*_*i*(*t*, *s*)_ is the NCD death parameter of country *i*, contained in the cell defined by country type *t* and development stage *s*, which is explained by the tree; *β*_0_ is the mean NCD death parameter across all group-level units (i.e. country type and development stage), *u*_*t*_ is the effect of country type *t*, *u*_*s*_ is the effect of development stage *s*, and *e*_*i*(*t*, *s*)_ is the country-level residual error term for city *i* contained in the cell defined by country type *t* and development stage *s*.

### Geodetector method

This method involves weight allocation to the two indicators in the calculation of the country development stage. The country development stage was calculated by urbanization and neonatal mortality rates, and the weight of each indicator was determined according to the intensity of the interpretation of the explained variables. Here, we used the *q*-statistic of the Geodetector to measure the intensity of interpretation, which is mainly used to detect the difference of geographic elements and their factors influencing the spatial distribution of research objects [56].

The *q*-statistic of the Geodetector can be used to explain the explanatory power of the influencing factors on the research object. The higher the *q*-value, the higher the explanatory power. In this study, the magnitude of the *q*-value of the Geodetector was used to judge the explanatory power of second trunks influencing factors that affect the country development stage in order to allocate a weight ratio and synthesize the final comprehensive development stage. The model formula is as follows:

2 $$ \mathrm{q}=1-\frac{1}{\mathrm{N}{\upsigma}^2}{\sum}_{\mathrm{h}=1}^{\mathrm{L}}{\mathrm{N}}_{\mathrm{h}}{\upsigma}_{\mathrm{h}}^2 $$

where *q* is the detection force indicator of the influencing factors of NCD deaths; *N*_*h*_ is the number of sample units in the secondary region; *N* is the number of sample units in the entire area; *L* is the number of secondary areas; *σ*^2^ is the variance of NCD deaths in the entire region; and ${\sigma}_h^2$ is the variance of the secondary area. The value range of *q* is [0, 1]. When *q* = 0, the explanatory power of the explanatory variable to the interpreted variable is 0, and the higher the *q*-value, the higher the explanatory power of the explanatory variable to the explained variable.

## Results

### Country-type geographic distribution

**Fig. 2:**
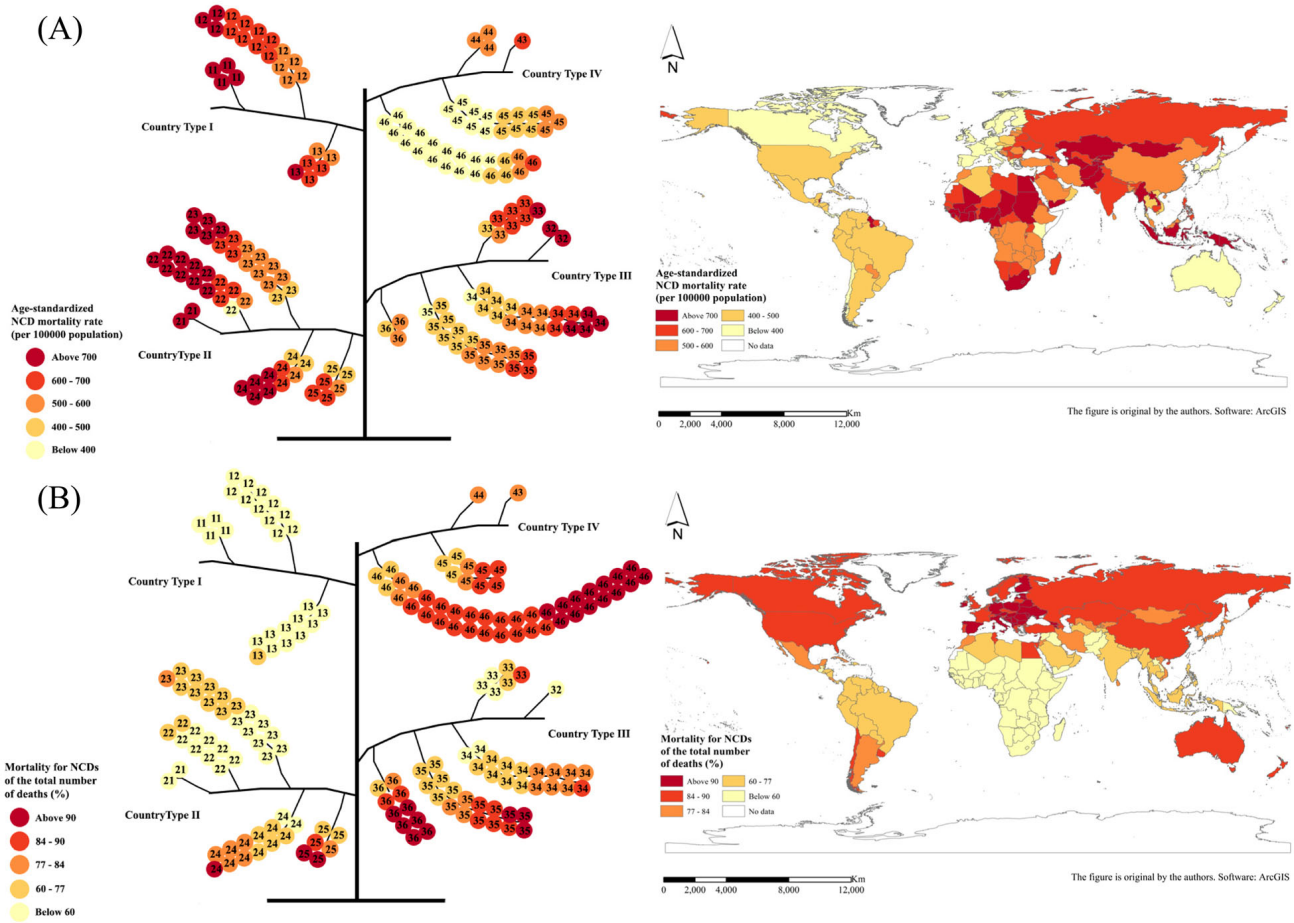
Global NCD evolution tree corresponding to the geospatial distribution: **a** 2015 age-standardized NCD mortality rate (per 100,000 population) and **b** 2015 mortality for NCDs of the total number of deaths (%). (NCDs include cancer, diabetes mellitus, cardiovascular diseases, digestive diseases, skin diseases, musculoskeletal diseases, and congenital anomalies)

After classification calculation, the global NCD death indicator evolution tree was constructed, and the tree structure is shown in Fig. 2. From the distribution of NCD deaths shown in the map, it can be seen that the NCD death index did not show a very significant spatial aggregation and other distribution rules in space, so the evolution tree model was used to explore the dimension of attributes. In addition, the distribution of NCD death indexes in different branches of the tree structure can be further explored.

### Comparison and trend of NCD death indexes for different country types

**Fig. 3:**
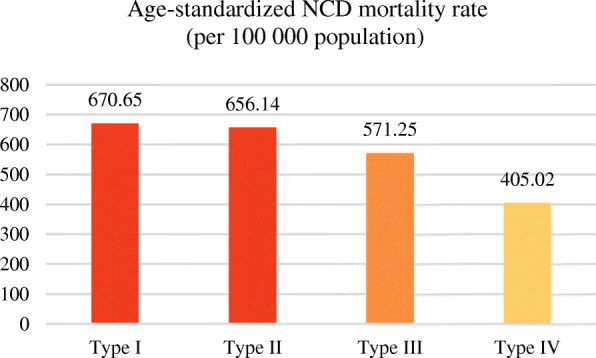
Average age-standardized NCD mortality rate (per 100,000 population) for different country types in 2015. (NCDs include cancer, diabetes mellitus, cardiovascular diseases, digestive diseases, skin diseases, musculoskeletal diseases, and congenital anomalies)

**Fig. 4:**
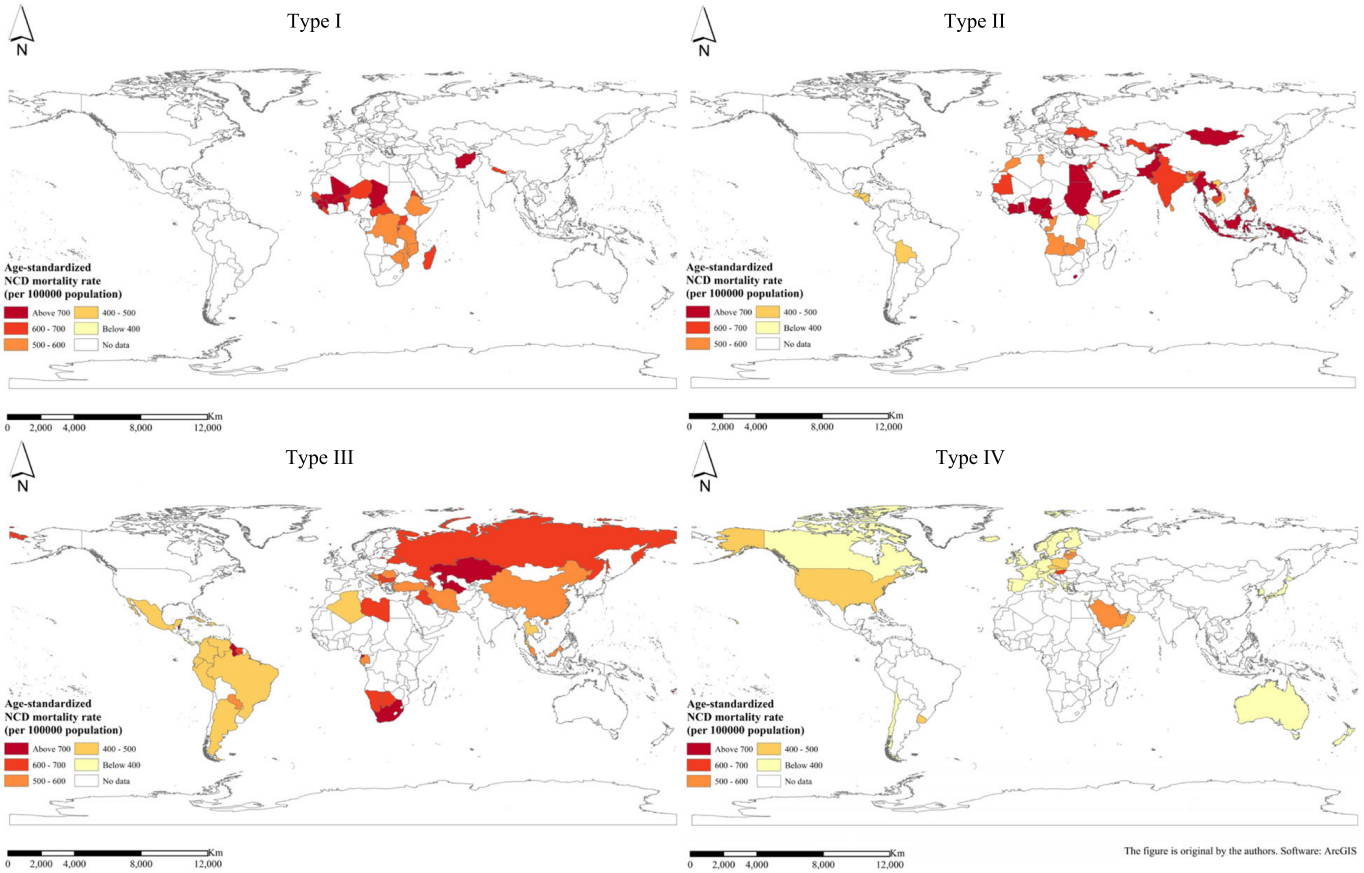
Spatial distribution of average age-standardized NCD mortality rate (per 100,000 population) for different country types in 2015. (NCDs include cancer, diabetes mellitus, cardiovascular diseases, digestive diseases, skin diseases, musculoskeletal diseases, and congenital anomalies)

The NCD death index presents different distribution patterns and development trends in different country types. As for the NCD mortality rate, as the national income increases, the mortality rate shows a gradual decline. The order of the average NCD mortality rate from high to low is as follows: low-income countries, lower middle-income countries, upper middle-income countries and high-income countries (Fig. 3). Figure 4 shows the geospatial distribution.

**Fig. 5:**
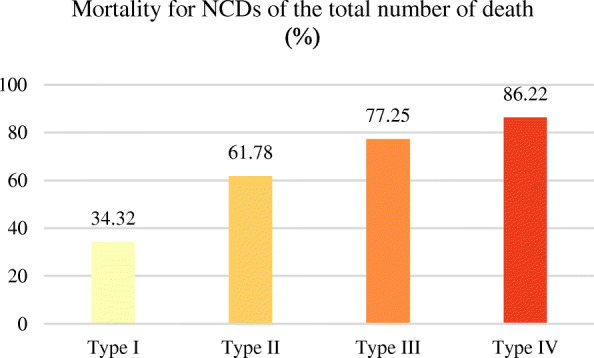
The mortality for NCD deaths of the total number of deaths (%) for different country types in 2015. (NCDs include cancer, diabetes mellitus, cardiovascular diseases, digestive diseases, skin diseases, musculoskeletal diseases, and congenital anomalies)

**Fig. 6:**
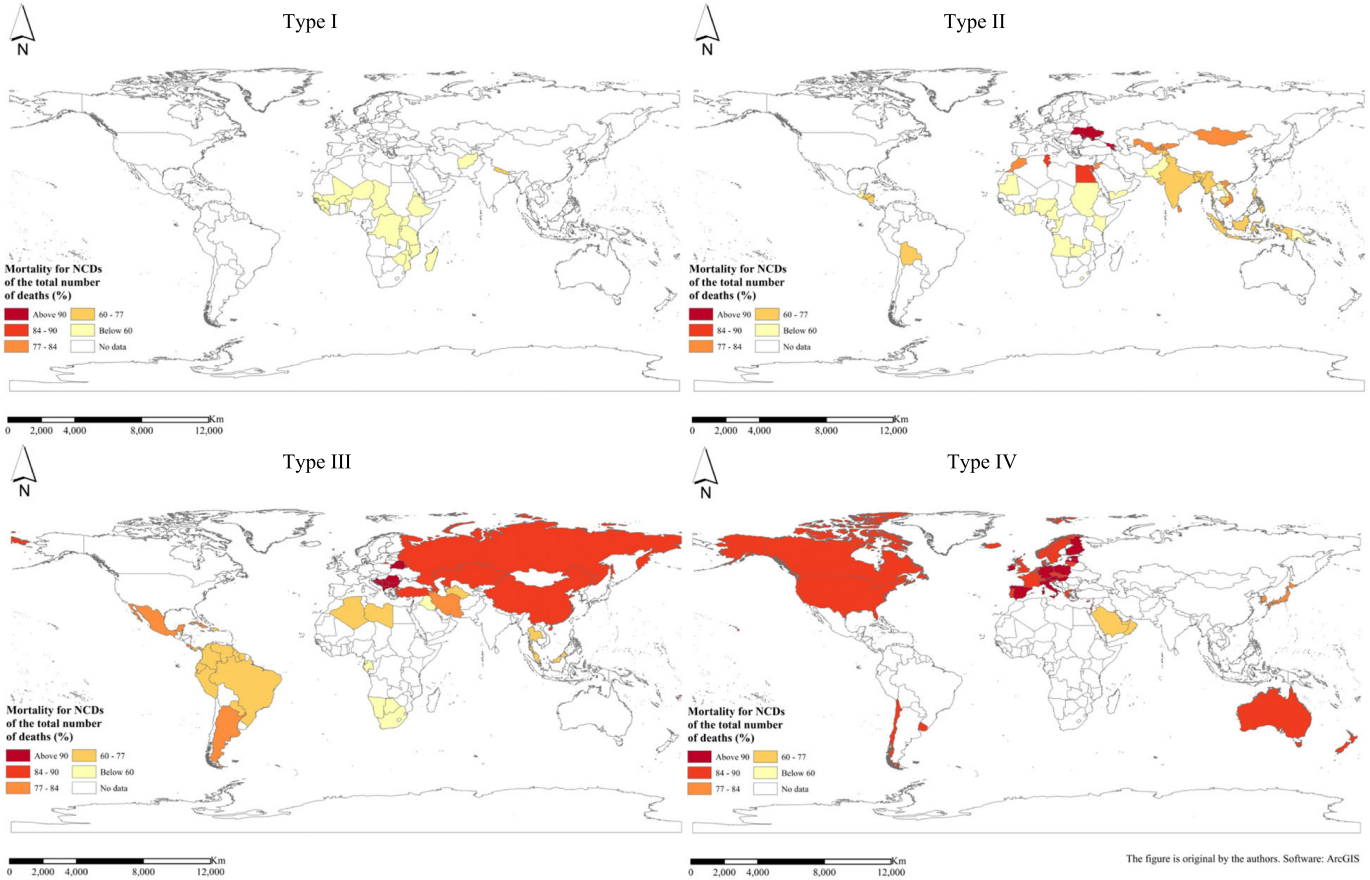
Spatial distribution of average mortality for NCDs of the total number of deaths (%) for different country types in 2015. (NCDs include cancer, diabetes mellitus, cardiovascular diseases, digestive diseases, skin diseases, musculoskeletal diseases, and congenital anomalies)

As regards the proportion of NCD deaths to total deaths, it shows an upward trend as the country’s income increases. That is, the proportion of average NCD deaths to total deaths from low to high is low-income countries, lower middle-income countries, upper middle-income countries and high-income countries (Fig. 5). Figure 6 shows the geospatial distribution.

### Comparison and trend of NCD death indexes at different country development stages

**Fig. 7:**
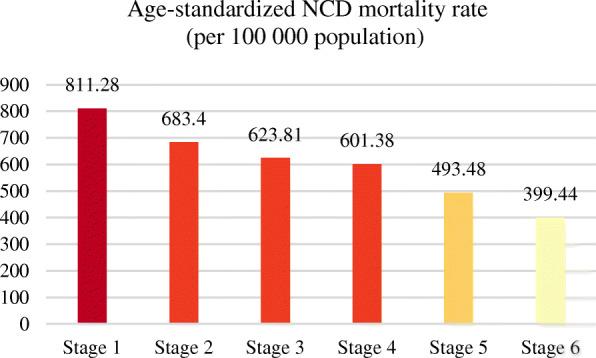
Average age-standardized NCD mortality rate (per 100,000 population) for different development stages in 2015. (NCDs include cancer, diabetes mellitus, cardiovascular diseases, digestive diseases, skin diseases, musculoskeletal diseases, and congenital anomalies)

**Fig. 8:**
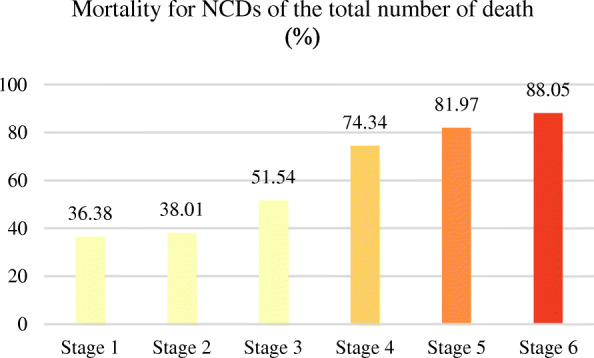
Average mortality for NCD deaths of the total number of deaths (%) for different development stages in 2015. (NCDs include cancer, diabetes mellitus, cardiovascular diseases, digestive diseases, skin diseases, musculoskeletal diseases, and congenital anomalies)

The countries were classified according to their socio-economic development stages by measuring the neonatal infant mortality and level of urbanization, and the NCD death indicators in different countries at different development stages also showed different distribution laws and trends. According to calculation and analysis, the NCD mortality rate gradually decreased with the increase of social and economic development stage (Fig. 7). In contrast, the proportion of NCD deaths to total deaths increased with the rise of socio-economic development (Fig. 8).

### Multilevel modelling and prediction

Using the hierarchical results of the evolution tree model and the principle of multilevel modelling (MLM) for model fitting, a prediction model of NCD death index was constructed, which was used to predict the future trend of NCD deaths. In MLM fitting, 70% of the records were extracted from all data to fit the MLM. The remaining 30% were used to verify the accuracy of the MLM. The predicted value of the calculated explained variable was plotted against the original observation value, and the accuracy of the MLM fitting was judged by the magnitude of the *R*^2^-value.

**Fig. 9:**
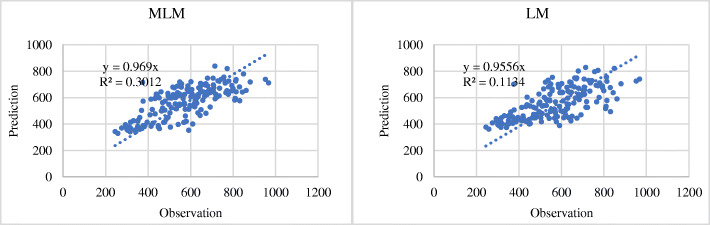
Accuracy comparison between multilevel model fitting results and linear regression model fitting results for age-standardized NCD mortality rate (per 100,000 population). (NCDs include cancer, diabetes mellitus, cardiovascular diseases, digestive diseases, skin diseases, musculoskeletal diseases, and congenital anomalies. In the figure, the horizontal axis represents the observation value of NCDs deaths, and the vertical axis represents the prediction value)

**Fig. 10:**
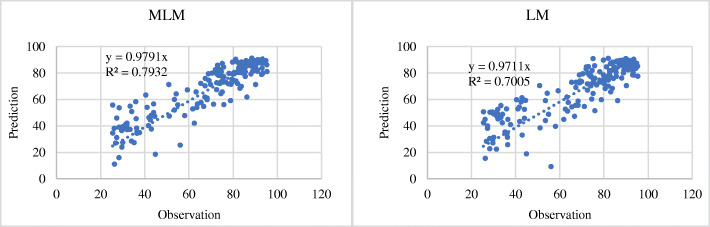
Accuracy comparison between multilevel model fitting results and linear regression model fitting results for mortality for NCDs of the total number of deaths (%). (NCDs include cancer, diabetes mellitus, cardiovascular diseases, digestive diseases, skin diseases, musculoskeletal diseases, and congenital anomalies. In the figure, the horizontal axis represents the observation value of NCDs deaths, and the vertical axis represents the prediction value)

At the same time, to compare whether the accuracy of the MLM fitting was better than that of the ordinary global model fitting, a linear regression model fitting experiment was also performed, and the *R*^2^-value was compared with the accuracy of the MLM. The specific comparison results are shown in Figs. 9 and 10. The *R*^2^-value of the MLM fitting results for NCD mortality rate was 0.30, whereas the *R*^2^-value of the linear model (LM) fitting results was 0.11 (Fig. 9). As regards the proportion of NCD deaths to total deaths, the *R*^2^-values of the MLM and LM fitting results were 0.79 and 0.70, respectively (Fig. 10). It was found that the fitting accuracy of the MLM is generally higher than that of the ordinary global linear regression model.

**Fig. 11:**
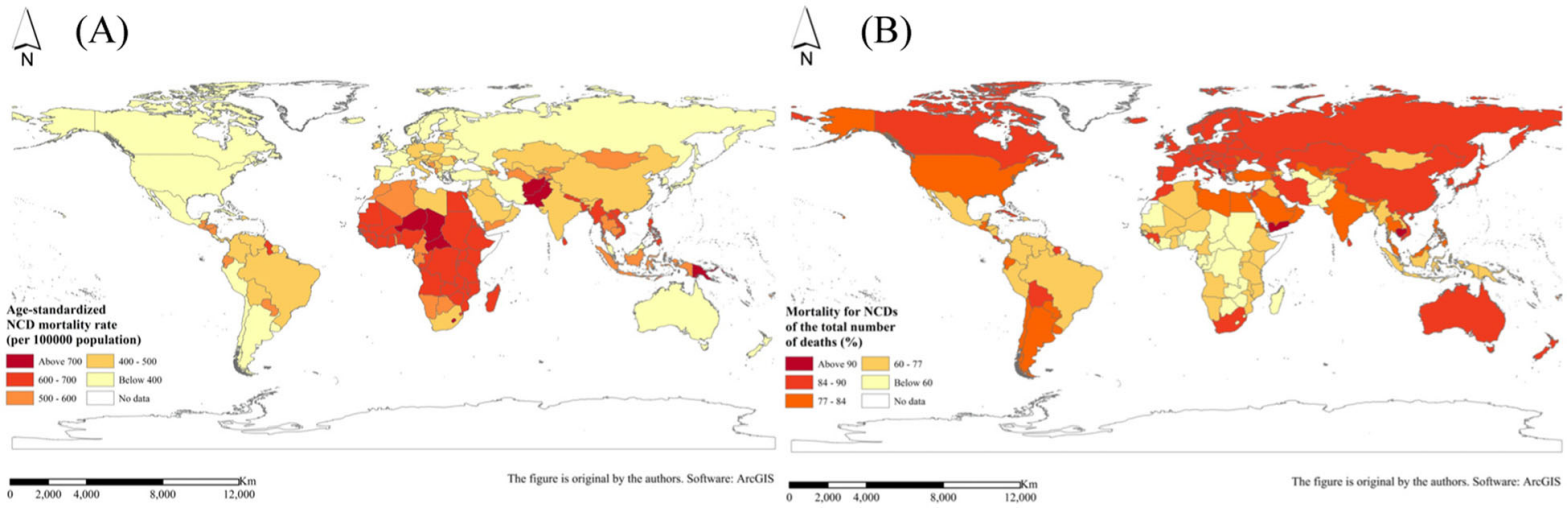
Spatial prediction of NCD death indexes in 2030: **a** global age-standardized NCD mortality rate (per 100,000 population) and **b** global mortality for NCDs of the total number of deaths (%). (NCDs include cancer, diabetes mellitus, cardiovascular diseases, digestive diseases, skin diseases, musculoskeletal diseases, and congenital anomalies)

Using this feature and advantage of the MLM, this study predicted the NCD death indicators in 176 countries around the world for 2030. The specific results are shown in Fig. 11. It has been predicted that by 2030, the global average age-standardized NCD mortality rate would be 510.54 (per 100,000 population) and the global average NCD deaths of the total number of deaths would be 75.26%.

## Discussion

In this study, the level of national income was selected as a classification indicator of the first branches of the NCD evolution tree. The level of income (or GDP) is one of the most direct factors for measuring a country’s economic development, and it is also a universal standard for international classification of countries. In the construction of the secondary trunks of the evolution tree, this study opted to use the level of urbanization and neonatal mortality as the calculation indicators, mainly because the development of urban areas and the improvement of medical conditions are also two important aspects to measure the social and economic development of a country. Some studies have found that, with the development of urbanization, the diet structure tends to be high in sugar, salt and oil, there is lack of exercise, and people tend to lead a sedentary life. Their average body mass index (BMI), blood lipids, blood pressure and other physical indicators also rise, eventually leading to the increased NCD incidence [57–62]. As the main component of chronic diseases, NCDs are closely related to socio-economic development. Therefore, this study focused on the influence of socio-economic factors and combined the perspective of the evolution tree to explore the law of NCD deaths. In addition, an important feature of Geotree is “evolution”. The Geotree classification indicators selected in this study—such as the level of national income, urbanization rate and neonatal infant mortality rate—are all development indicators that measure a country’s level of development from three aspects: (i) economic, (ii) social and (iii) medical. The basic socio-economic factors are also easy to obtain, which is convenient for the subsequent evolution modelling of future NCD death index trends.

Moreover, the interpretation power of the two-level classification results of Geotree was also calculated using the Geodetector. The specific results are shown in Table 3. The *q*-values of the country types reached 0.45 (age-standardized NCD mortality rate) and 0.61 (NCD deaths of the total number of deaths), and both passed the significance test. It has been proved that the classification of the country type has a strong interpretation of the NCD death indexes.

**Table 3:** *q*-Values of the country types for different NCD death indexes. (NCDs include cancer, diabetes mellitus, cardiovascular diseases, digestive diseases, skin diseases, musculoskeletal diseases, and congenital anomalies)

	Age-standardized NCD mortality rate	Mortality for NCDs of the total number of deaths
*q* statistic	0.452723	0.612019
*p* value	0.000	0.000

The national socio-econamic development stage also maintained a high level of explanation for NCD mortality (with a *q*-value of 0.49, passed the significance test) and the proportion of NCD deaths to total deaths (with a *q*-value of 0.74, passed the significance test; Table 4).

**Table 4:** *q*-Values of the national socio-economic development stage for different NCD death indexes. (NCDs include cancer, diabetes mellitus, cardiovascular diseases, digestive diseases, skin diseases, musculoskeletal diseases, and congenital anomalies)

	Age-standardized NCD mortality rate	Mortality for NCDs of the total number of deaths
*q* statistic	0.492107	0.735285
*p* value	0.000	0.000

Combining the results provided previously, it can be found that with the development of economy, no matter whether the level of income increases, or urbanization rate and medical level increase, the NCD mortality rate decreases with it. This shows that NCDs are effectively controlled with the level of socio-economic development. The improvement of the level of economic development allows more patients with NCDs to be able to afford the cost of treating the disease and choose to seek medical treatment [63], and the improvement of medical conditions allows NCDs to be effectively treated, and ultimately, the mortality rate is reduced.

In contrast, the proportion of NCD deaths to total deaths is increasing, which shows that with the development of economy, infectious diseases can be better controlled through the development of economy and improvement of medical conditions. However, the adjustment of industrial structure, change of lifestyle and change of dietary structure brought about by economic development will aggravate the NCD incidence and deaths. Studies have suggested that the occurrence of NCD will be more common in fast-growing cities with higher survival stress, fast-paced lifestyles, lack of exercise and rising air pollution [64–66]. The increase in the proportion of NCD deaths to total deaths with economic development also proves that the mechanism of NCDs is more complicated.

From the perspective of the branch structure of the evolution tree combined with geographic space, the distribution of the four country types is roughly as follows: country type I (low-income economies), mainly distributed in Africa; country type II (lower middle-income economies), mainly distributed in Southeast Asia, South Asia, Central Asia, other Asian regions and a small part of Africa; country type III (upper high-income economies), located in East Asia, Latin America, West Asia and North Africa, but also includes parts of South Africa and a small part of southern Europe; and country type IV (high-income economies), mainly North America, Europe and a small part of Oceania. On the contrary, the regions with higher NCD mortality rate are countries with lower levels of economic development, mainly countries in Africa and some countries in Asia (Southeast Asia, South Asia, etc.). The regions with a higher proportion of NCD deaths to total deaths are developed regions such as North America and Europe. This also proves from the side that the NCD death indexes and level of economic development show a high correlation; that is, the NCD mortality rate and the level of economic development generally show a negative correlation, and the proportion of NCD deaths to total deaths and the level of economic development are roughly positively correlated.

Although the close relationship between NCDs and socio-economic development shows a positive or negative correlation as a whole, the leading factors for the occurrence of NCDs are different in countries and regions at different socio-economic development stages. In comparison, in less developed areas (such as country type I and II), the residents have higher levels of alcohol and cigarette consumption and start smoking at a young age (including second-hand smoke); even tobacco consumption by some poor families is equivalent to the dietary expenditures of one or two undernourished children [36, 66–70]. These people also consume insufficient fruit and vegetables [16, 32, 66, 71–73]. Moreover, they may develop NCDs because of some congenital factors or the induction of a harsh living environment, such as poor breastfeeding and being underweight during childhood [74]. In addition, because of the backward level of socio-economic development, fossil fuels account for a relatively high proportion in household use, such as heating and cooking, so household air pollution is also the cause of some kinds of NCDs [74]. In contrast, the residents in regions with high levels of socio-economic development (such as country type III and IV) display lack of physical activities and consume more high-oil and high-salt processed foods [66, 75–77]. Therefore, in contrast to underdeveloped regions, NCDs caused by abnormal physical indicators (blood pressure, blood lipids, BMI, etc.) resulted from acquired dietary habits and living habits are the main part of NCD incidence [59]; this part also needs to be explored in future experiments.

In the MLM fitting experiment, an MLM and a general global linear regression model were used for fitting. From the calculation results, it can be seen that the prediction accuracy of the MLM is better than that of the general global linear regression model. This shows that there is hierarchical heterogeneity in the distribution of NCDs. If only the global linear regression model is used, although it is simple to calculate, it will ignore this characteristic, resulting in less accurate results than the MLM.

Regarding the current situation of non-communicable diseases gradually becoming popular in low- and middle-income countries and developing countries in recent years, governments of all countries in the world should pay sufficient attention and take effective measures to prevent the prevalence of non-communicable diseases. The government can spread the risk factors and prevention methods of diseases to the public by introducing more authoritative NCDs popularization and prevention measures, so as to arouse ordinary people’s higher attention. At the same time, vigorously carrying out public health monitoring and improving the health management system are also effective measures to prevent and control non-communicable diseases. Under the guidance of medical theory, through the establishment of health records to conduct a scientific analysis of the public’s health status, targeted expert advice and management programs are given. In addition, the evolutionary tree method can also be used to calculate the relationship between the level of socioeconomic development and NCDs distribution in different regions of a country. Based on the simulation results, the government can allocate medical resources and public health facilities in a targeted manner to improve the utilization of medical resources. Countries can also establish international organizations that collaborate in the prevention and control of diseases, learn from each other’s useful experience, and provide some assistance to countries with relatively backward medical standards to jointly contribute to the prevention and control of non-communicable diseases.

This article explores the distribution and evolution of NCD death indicators from the socioeconomic dimension. NCD deaths have obvious stratified heterogeneity, showing different distribution patterns in countries and regions with different social and economic conditions. The evolution tree model is a classification-based visualization method. Its core is stratified heterogeneity and the calculation process is not limited by the data dimension, which can cover as many factors as possible that affect the development of things. Exploring the distribution and evolution of NCDs death on a hierarchical basis can effectively control some confounding factors that exist in ordinary global studies and obtain more accurate results. And the perspective of this study is between macro and micro, which is more conducive to playing an important guiding role in the allocation of medical resources and the implementation of prevention and control measures. Moreover, countries with different levels of development can also have a higher reference value for their NCDs prevention and control by referring to the development and prevention measures of countries on the same branch and trunk as their own.

However, this study also has certain limitations. Limited to the lack of indicators of global socio-economic factors that use countries as statistical units, this article can only use common public data indicators to do the evolution tree classification, which to a certain extent weakens the advantages of the evolution tree’s multi-dimensional coordinate system. In the follow-up research, this is an aspect that needs to be improved. Enrich the types of indicators of social and economic factors as much as possible, so that the advantages of the evolution tree model can be better played, and the research results can be more detailed and comprehensive.

## Conclusions

This study found that there is a clear correlation between socio-economic factors and NCD death indicators and that there is significant stratified heterogeneity in the distribution of NCD deaths. The construction of the spatio-temporal evolution tree model is based on a country’s recognition of its own level of NCD deaths, helping it to have a more comprehensive positioning of the characteristics of the NCD death indicator in the socio-economic dimension. By grading the branches of the evolution tree, different country types can find their place in the tree structure. Countries located on the same first branch belong to the same type. They have similar socio-economic conditions and similar development paths. Therefore, the development of secondary branches from the lower to higher stage on the same primary branch is the common development path of the same city types. By grading the branches of the evolution tree, different country types can find their place in the tree structure. Countries on the same branch belong to the same type, and the secondary trunks under each branch represent the development status at different stages. The socio-economic factors of the countries on the same branch are similar, and they generally follow the rule of development from lower secondary trunks to the higher ones; that is, they have similar development paths. When a country finds its own position in the tree-like coordinate system of the evolution tree, it can refer to the development direction of the country at a more advanced stage and combine its experience in disease prevention and control to locate its own development goals and formulate corresponding NCD prevention and control plans. Different country types can also learn from each other and take the road of NCD prevention and control in accordance with their own national conditions on the basis of their own characteristics.

## Data Availability

All data needed to evaluate the conclusions in the paper are present in the paper. Additional data related to this paper may be requested from the authors.

## Abbreviations

BMI: Body mass index
GDP: Gross domestic product
GNI: Gross national income
LMICs: Low- and middle-income countries
MLM: Multilevel model
LM: Linear model
NCDs: Non-communicable diseases
WHO: World Health Organization
